# Neural correlates of interference resolution in the multi-source interference task: a meta-analysis of functional neuroimaging studies

**DOI:** 10.1186/s12993-018-0140-0

**Published:** 2018-04-10

**Authors:** Yuqin Deng, Xiaochun Wang, Yan Wang, Chenglin Zhou

**Affiliations:** 1 Department of Sport Psychology, School of Kinesiology, Shanghai University of Sport, 399 Chang Hai Road, Shanghai, 200438 People’s Republic of China; 2 Interdisciplinary Center for Social and Behavioral Studies, Dongbei University of Finance and Economics, Dalian, 116025 Liaoning Province People’s Republic of China

**Keywords:** Interference resolution, Multi-source interference task, Functional magnetic resonance imaging, Meta-analysis

## Abstract

**Background:**

Interference resolution refers to cognitive control processes enabling one to focus on task-related information while filtering out unrelated information. But the exact neural areas, which underlie a specific cognitive task on interference resolution, are still equivocal. The multi-source interference task (MSIT), as a particular cognitive task, is a well-established experimental paradigm used to evaluate interference resolution. Studies combining the MSIT with functional magnetic resonance imaging (fMRI) have shown that the MSIT evokes the dorsal anterior cingulate cortex (dACC) and cingulate–frontal–parietal cognitive-attentional networks. However, these brain areas have not been evaluated quantitatively and these findings have not been replicated.

**Methods:**

In the current study, we firstly report a voxel-based meta-analysis of functional brain activation associated with the MSIT so as to identify the localization of interference resolution in such a specific cognitive task. Articles on MSIT-related fMRI published between 2003 and July 2017 were eligible. The electronic databases searched included PubMed, Web of Knowledge, and Google Scholar. Differential BOLD activation patterns between the incongruent and congruent condition were meta-analyzed in anisotropic effect-size signed differential mapping software.

**Results:**

Robustness meta-analysis indicated that two significant activation clusters were shown to have reliable functional activity in comparisons between incongruent and congruent conditions. The first reliable activation cluster, which included the dACC, medial prefrontal cortex, supplementary motor area, replicated the previous MSIT-related fMRI study results. Furthermore, we found another reliable activation cluster comprising areas of the right insula, right inferior frontal gyrus, and right lenticular nucleus-putamen, which were not typically discussed in previous MSIT-related fMRI studies.

**Conclusions:**

The current meta-analysis study presents the reliable brain activation patterns on MSIT. These findings suggest that the cingulate-frontal-striatum network and right insula may allow control demands to resolve interference on MSIT. These results provide new insights into the neural mechanisms underlying interference resolution.

## Background

The interjection of goal-irrelevant information with goal-relevant information is referred to as cognitive interference. For instance, while trying to concentrate on your job, you may have to inhibit the habitual tendency to check your Facebook feed. Successful interference resolution depends on flexible cognitive control that suppresses goal-irrelevant inputs, while selecting and organizing goal-relevant inputs.

The multi-source interference task (MSIT) is a cognitively demanding well established paradigm for assessment of cognitive interference. In the MSIT, stimuli (e.g., the digits “1”, “2”, or “3”, or a letter “X” or a digit “0”) are organized into groups of three and participants are required to recognize a unique target among the three items under congruent and incongruent conditions [1, 2]. The spatial position of the unique target matches its correct button-press response in the **congruent condition** (e.g., “1XX” or “100”, the unique targets were “1” and the button was responded at the 1st position) and is in conflict with its correct button-press response in the **incongruent condition** (e.g., “331”, the unique targets were “1” but the button was responded at the 3nd position). In the MSIT, an interference effect is indexed by the difference in reaction time between incongruent and congruent conditions.

In an initial pilot imaging study of MSIT performance, Bush et al. reported that the dorsal anterior cingulate cortex (dACC) was reliably activated at either the individual- or group-level in the incongruent condition, compared with congruent condition, indicating that the dACC is important for interference processing [1]. Likewise, imaging studies with both youth and adults have shown increased activation in the dACC during MSIT performance and such dACC activity correlated with interference- and error-processing [3, 4]. Moreover, studies examining female twins [5] and subjects diagnosed with attention deficit hyperactivity disorder (ADHD) [6] have provided the evidence indicating that MSIT-related dACC activation may be attributable to genetic factors. Clinical studies have associated dACC dysfunctions with MSIT-related cognitive interference in patients with pediatric obsessive–compulsive disorder (OCD) [7], schizophrenia [8], and posttraumatic stress disorder (PTSD) [9, 10], suggesting that dACC abnormalities may contribute to cognitive difficulties.

Cingulate-frontal-parietal (CFP) cognitive-attentional networks have also been reported to be widely and significantly activated by MSIT [11, 12]. In a sample of younger and older adults, interference process on MSIT was associated with activation of the fronto-parietal and basal ganglia networks [13]. Patients with ADHD have been reported to show dysfunction of CFP cognitive-attention networks and abnormal ACC activity during interference processing [14, 15]. Also, relative to healthy controls, patients with chronic low back pain have been reported to have decreased MSIT-related activation in structures of the CFP network, including the dorsolateral prefrontal cortex, dACC, and superior parietal cortex [16]. Patients with OCD have been reported to exhibit functional abnormalities in the cingulate-frontal circuits, insular cortex and the putamen when performing the MSIT [17–19]. These findings could help to explain the inhibitory control deficits in OCD.

The MSIT interference effects on cortical activity in the aforementioned studies were variable, perhaps due to differences in study design and sample characteristics. Hence, a quantitative assessment of brain network activity in MSIT is needed. In the present study, we applied a meta-analytic approach to synthesize the published MSIT-fMRI studies with the aim of clarifying the locations of generators of interference processing during MSIT performance. We used effect-size signed differential mapping (ES-SDM) as the meta-analytic toolbox [20–22]. The ES-SDM is a reliable quantitative voxel-based meta-analytic method, which allow to integrate statistical parametric maps and peak coordinates. The meta-analytic method has to be superior to other coordinate-based meta-analytical methods owing to its ability to enable reconstruction of both positive and negative coordinate in the same map, leading to a signed differential map and keeping a special voxel from wrongly arising to be positive and negative at the same time [23]. It provides Jackknife sensitivity and heterogeneity analyses to further confirm the replicability of voxel-based meta-analytic findings. In this meta-analysis, we expected to demonstrate replicable brain activation patterns associated with MSIT interference processing within the dACC and in the CFP network.

## Methods

### Data sources and study selection

**Fig. 1:**
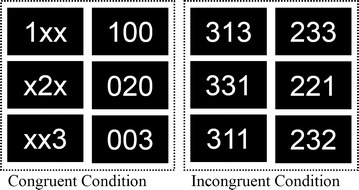
Illustration of the stimuli in multiple-source interference task (MSIT). Example congruent (right) and incongruent (left) condition trials. Participants were required to recognize the unique target among three items. In the *congruent condition*, the spatial position of the unique target matches the correct button-press response (e.g., “1XX” or “100”, the unique target “1” and its response button are both in the 1st position). In the *incongruent condition*, the spatial position of the unique target is in conflict with its correct button-press response (e.g., in “331”, the unique target “1” is in the 3rd position while its response button is in the 1st position)

We conducted a systematic search of PubMed (http://www.pubmed.org), Web of Knowledge (http://apps.webofknowledge.com), and Google Scholar (http://scholar.google.com) for MSIT-related fMRI studies from 2003 to July 2017. The search term combinations used were: “multi-source interference task” and “functional magnetic resonance imaging”. A total of 603 papers were found and assessed to determine if they met the following criteria: (1) an original article published in a peer-reviewed English-language journal; (2) a study employed MSIT during fMRI with a healthy control group; (3) the BOLD fMRI technique was used; (4) MSIT stimuli were numbers and MSIT trials included both incongruent and congruent conditions, as in Fig. 1; (5) the fMRI data were analyzed by contrasting of incongruent versus congruent conditions; (6) a whole-brain, voxel-wise analysis was applied in the fMRI data analysis; and (7) fMRI activation clusters were reported in Talairach or MNI coordinates.

### Data analysis

#### Voxel-wise meta-analysis

Differential BOLD activation patterns between the incongruent and congruent condition were meta-analyzed in Anisotropic Effect-Size Signed Differential Mapping (ES-SDM) software, version 4.13 (http://www.sdmproject.com). ES-SDM, which is a voxel-based meta-analytic approach, is described in detail in the SDM tutorial and publications (http://www.sdmproject.com/software/tutorial.pdf) [20, 22, 23].

The meta-analysis procedure followed three steps. First, the peak coordinates of brain activation differences between incongruent and congruent conditions were retrieved from each study. Peak coordinates were recorded with their *z*-values, where *z* could be a positive *z*-statistic or a negative *z*-statistic. Second, effect-size and effect-size-variance maps were recreated for each study. Anisotropic kernels were used to optimize the accuracy of these maps [22]. Activation maps, both with contrast of incongruent > congruent conditions, and contrast of congruent > incongruent conditions were calculated by SDM [21]. Third, a voxel-wise random-effects meta-analysis that considered sample size, intra-study variance, and inter-study heterogeneity was conducted [20, 22]. The statistical significance was evaluated with a voxel-level (height) threshold of *p* < 0.00001 and a cluster-level (extent) threshold of k = 100 voxels [20].

#### Complementary analyses

To evaluate the robustness and replicability of meta-analytic results, whole-brain-voxel-based Jackknife sensitivity analysis was conducted, wherein the same meta-analysis is repeated nine times, each time with a different single study excluded. The principle of the procedure is that if the previous meta-analytic results remain significant, the results can be considered robust and reliable [20, 23]. Statistical significance was set based on the same thresholds applied in the voxel-wise meta-analytic results.

Employing a random effects model with Q statistics, we analyzed heterogeneity to determine whether the observed inter-study variance was larger than that resulting from sampling error alone [20, 23]. Such analyses can reveal any false-positive brain regions due to significant unexplained between-study variability. The default ES-SDM thresholds were set for the heterogeneous results based on a voxel-level threshold of *p* < 0.005 and a cluster-level threshold of k = 10 voxels [20].

A subgroup analysis of adult samples was conducted to examine if potential confounding effects of age contributed to the heterogeneity of the findings [20, 23]. To evaluate the replicability of meta-analytic results, the statistical significance of the subgroup analysis was also identified with the same thresholds applied in the voxel-wise meta-analytic results.

## Results

### Characteristics of the cohorts of the studies included for meta-analysis

In total, 20 studies met the inclusion criteria for our meta-analysis, of which 12 were excluded for overlapping or duplicating data, leaving eight studies eligible for the final meta-analysis. One of these involved two different healthy sample populations (an adult sample and a youth sample), and the interference effect results of each of the two samples were treated as an independent dataset in the meta-analysis [3]. Hence, our meta-analysis consisted of “nine” study datasets [1–3, 8, 24–27]. The detailed demographic and task-related variables of each study are presented in Table 1.

**Table 1:** Summary of MSIT-fMRI studies (8 studies, 9 datasets) included in the meta-analysis

Study	Adults	Subjects, n (female, n)	Ages	Testa	Software	FWHM	Threshold	Interference condition	Control condition	Effect-size *d*
Bush et al. [1]	1	8 (4)	30.4 ± 5.6	3	AFNI	NA	Corrected	787 ± 129	479 ± 92	2.75
Fitzgerald et al. [3] adult	1	21 (6)	39.8 ± 9.4	3	SPM2	NA	Corrected	1044 ± 193	803 ± 197	1.24
Fitzgerald et al. [3] youth	0	23 (12)	13.2 ± 3	3	SPM2	NA	Corrected	1062 ± 338	754 ± 212	1.09
Gianaros et al. [2]	1	97 (50)	40.1 ± 6.2	3	SPM8	6	Corrected	905 ± 199.6	540.4 ± 108.9	2.27
Heckers et al. [8]	1	15 (0)	46.6 ± 9.1	1.5	SPM99	8	Uncorrected	873 ± 79	603 ± 67	3.69
Kim et al. [24]	0	28 (14)	13.6 ± NA	3	SPM8	8	Corrected	969.8 ± NA	686.9 ± NA	NA
Shehzad et al. [25]	1	104 (0)	23.9 ± 5.2	3	FSL	6	Corrected	977.57 ± 135.41	632.81 ± 84.79	3.05
Weissman et al. [26]	1	24 (9)	21 ± NA	3	SPM8	8	Corrected	858 ± NA	661 ± NA	NA
Yücel et al. [27]	1	24 (11)	29.58 ± 6.45	3	FSL	5	Corrected	1190 ± 172	861 ± 162	1.97

The characteristics of the analyzed MSIT-fMRI studies (nine datasets) are summarized in Table 1. Altogether, data from a total of 344 subjects (106 females), with a mean age of 29.22 years. Among them, there were 293 adults (80 females; mean age, 31.97 years) and 51 youths (26 females; mean age, 13.42 years).

### Changes in regional brain responses to cognitive tasks in MSIT studies, complementary analyses

**Fig. 2:**
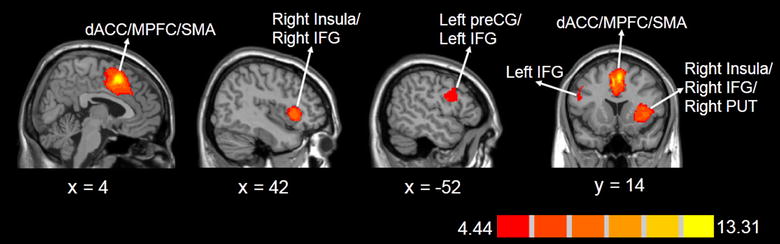
Significant functional brain activation for incongruent condition > congruent condition determined by meta-analysis. Results with *p* < 0.00001 (cluster size ≥ 100 voxels) are shown. The color bar indicates the regional value of the signed differential mapping (SDM) statistic. *dACC* dorsal anterior cingulate cortex; *MPFC* medial prefrontal cortex; *SMA* supplementary motor area; *IFG* inferior frontal gyrus; *PUT* putamen; *preCG* precentral gyrus

Voxel-wise meta-analysis showed that, compared to the congruent condition, the incongruent condition produced significantly increased activity in three clusters, involving the dACC, medial prefrontal cortex (MPFC), supplementary motor area (SMA), right insula, right inferior frontal gyrus (IFG), right lenticular nucleus-putamen (PUT), left precentral gyrus, and left IFG (Table 2, Fig. 2). As reported in Table 2, whole-brain jackknife sensitivity analysis revealed two significant clusters involving the dACC, MPFC, SMA, right insula, right IFG, right PUT were highly replicable across all nine datasets. Only three datasets had significantly activated clusters in the left precentral gyrus and left IFG in common.

**Table 2:** The main difference in activation between the incongruent and congruent conditions during multi-source interference task

Region	Brodmann area	Maximum			Cluster		Jackknife sensitivity analysis
		MNI coordinates x, y, z	SDM value	p value	Number of voxels	Breakdown (number of voxels)	
dACC/MPFC/SMA	6/8/24/32	4, 14, 48	13.305	~ 0	2056	Supplementary motor area (911) Median cingulate/paracingulate gyri (734) Anterior cingulate/paracingulate gyri (105) Superior frontal gyrus, medial (141) Median network, cingulum (59) *Corpus callosum* (106)	9 out of 9
R insula/R IFG/R PUT	11/45/47/48	42, 20, − 2	8.765	~ 0	902	R insula (366) R inferior frontal gyrus (207) R fronto-insular tract (26) R lenticular nucleus, putamen (136)	9 out of 9
L preCG/L IFG	6/44/48	− 52, 2, 22	6.335	~ 0	279	L precentral gyrus (135) L inferior frontal gyrus (82) L middle frontal gyrus (16)	3 out of 9

*L* left; *R* right; *MNI* Montreal Neurological Institute; *SDM* signed differential mapping; *dACC* dorsal anterior cingulate cortex; *MPFC* medial prefrontal cortex; *SMA* supplementary motor area; *IFG* inferior frontal gyrus; *PUT* putamen; *preCG* precentral gyrus

As reported in Table 3, our heterogeneity analysis detected a significant unexplained inter-study variance, focused mainly on the occipital lobe, parietal lobe, and right cerebellum. But the heterogeneity analysis did not reveal brain regions with significant incongruent versus congruent differences in voxel-wise meta-analysis results (Table 3). Significant clusters in the dACC, MPFC, SMA, right insula, right IFG, and right PUT, but not in the left precentral gyrus and left IFG, were retained in a subgroup analysis of adult subjects. Hence, significant clusters of activation in the voxel-wise meta-analysis results involving the dACC, MPFC, SMA, right insula, right IFG, and right PUT were reliable and robust.

**Table 3:** Heterogeneity analysis results

Regions	Brodmann area	Maximum MNI coordinates x, y, z	Voxels	SDM value	p value
R fusiform gyrus/R cerebellum	37	38, − 50, − 22	188	6.109	~ 0
R angular gyrus/R superior parietal gyrus/R superior occipital gyrus	7	26, − 62, 48	105	6.564	~ 0
L middle frontal gyrus/L precentral gyrus	6	− 28, − 8, 50	78	6.29	~ 0
L middle occipital gyrus	18/19	− 32, − 88, 16	72	5.962	~ 0
L inferior occipital gyrus/L inferior temporal gyrus	19/37	− 42, − 66, − 8	48	6.112	~ 0
R supramarginal gyrus/R inferior parietal gyri	2/40	44, − 38, 44	47	5.907	~ 0
R inferior occipital gyrus	19	40, − 78, − 4	40	5.534	~ 0
L middle occipital gyrus/L superior occipital gyrus	19/7	− 26, − 68, 32	38	6.145	~ 0
L inferior occipital gyrus/L middle occipital gyrus	19	− 34,− 86,− 6	37	5.962	~ 0
R middle occipital gyrus	19	34, − 68, 30	23	5.920	~ 0
L anterior thalamic projections		− 12, − 16, 2	18	5.349	~ 0
L postcentral gyrus/L inferior parietal gyrus	2	− 48, − 34, 52	15	5.478	~ 0

*L* left; *R* right; *MNI* Montreal Neurological Institute; *SDM* signed differential mapping

## Discussion

To our knowledge, this is the first report of a voxel-based meta-analysis that identified MSIT-associated functional brain activation. Robustness analyses confirmed that the significance of two major activation clusters involving the dACC, MPFC, SMA, right insula, right IFG, and right PUT was reliable and robust during comparison between incongruent and congruent conditions.

Our findings are consistent with previous fMRI studies on MSIT indicating robust activation in the dACC, MPFC and SMA during interference processing when incongruent and congruent conditions are compared [1, 11, 28]. In the MSIT, subjects need to respond to the target while ignoring simultaneously presented unrelated information. Conflict is generated when the task-irrelevant information is incompatible with the target, thereby impeding the processing of task-relevant information. The dACC is recruited to monitor conflict. Higher dACC activity for incongruent trials has also been found in the flanker task [29, 30], Stroop task [30, 31], and Simon task [30, 32], providing further evidence for the supposition that the ACC is involved in detecting conflict in various interference tasks. Electrophysiological studies in both humans and monkeys have shown that dACC neurons firing rates increase during conflict processes and this increase is thought to promote ongoing behavioral adjustment [33–36]. Moreover, our findings are consistent with the conflict-monitoring hypothesis, which posits that increased ACC activity occurs when a high level of conflict is detected in incongruent trials, thereby recruiting top-down cognitive modulation to resolve the conflict and improve performance [37]. On the other hand, most imaging studies examining MSIT performance have found higher SMA activity in incongruent trials than in congruent ones and our meta-analysis results confirmed this conclusion. Anatomically, the SMA has ventral connections with the dACC [38]. Anatomically, the SMA has ventral connections with the dACC [38]. Thus, the SMA and dACC might work together to solve the interference challenge in the MSIT. Functionally, the SMA participates in movement planning and in action initiation and inhibition [38–40]. In other conflict tasks, researchers have also found that the SMA played a leading role in guiding the process of action-monitoring [41]. In a recent review of neuroimaging, electrophysiological, and stimulation studies of the SMA, Coull et al. proposed that the SMA may be involved in the cognitive development of a sensory representation of time, in addition to its aforementioned roles [42]. Altogether, the SMA is implicated in the process of deciding when to initiate an action or not. This possibility is supported by a prior electrophysiological study showing that neuronal activity in the SMA is associated with proactive and reactive behavioral control in a stop-signal task [43]. The SMA plays a proactive role in controlling arm movements to regulate motor readiness, and is involved in inhibiting arm movements in response to an unexpected stop signal. Accordingly, in the MSIT, after conflict is detected by the dACC, the SMA might be activated to plan movements and to establish flexible adaptive behavior.

An unexpected finding in our meta-analysis was a significantly active cluster involving the right IFG, right insula, and right PUT in comparisons between incongruent and congruent conditions. But previous studies employing the MSIT have found that CFP cognitive-attentional networks are reliably activated under these conditions. Although the result was not predicted, it is in agreement with a previously proposed role of the right IFG [44]. In a systematic review of a decade of literature regarding right IFG functions, Aron et al. found that the right IFG, together with one or more fronto-basal-ganglia network regions (including the PUT), may play a critical role in outright action-stopping in response to external stop or salient signals or internal goals [44]. The authors of other reviews of empirical electrophysiological and neuroimaging data from various inhibition paradigms (e.g., Stroop, Simon, and flanker tasks) have proposed that right IFG/basal ganglia pathways may contribute to goal-directed and habitual inhibition [45–47]. However, Bari and Robbins, who contributed a systematic summary of inhibition and impulsivity studies, suggested that the right IFG appears to be involved not only in the processing of response inhibition but also in the updating of goal-related plans of action [48]. According to these reviews, incongruent MIST trials produce more interference and inhibitory control than congruent trials due to the need to suppress distracting stimuli. Thus, interference may be resolved by engagement of the right IFG and PUT.

The insula is a commonly activated region in the go/no-go task, flanker task, and stimulus–response compatibility task, and insula activation has been shown to be related to interference resolution in each task [49]. Cai et al. examined causal interactions within core frontal-cingulate-parietal regions in the stop-signal task and the flanker task [50]. The strength of causal interaction between the right anterior insula and dACC was found to be greater under high cognitive control conditions than under low ones, and to be significantly associated with cognitive control ability indices in both the stop-signal task and the flanker task, suggesting that both the right anterior insula and dACC may be involved in cognitive control in various interference tasks. On the other hand, the insula and dACC are constituents of “salient network”, in which the right insula is thought to detect salient stimuli for recruitment of inhibitory control [51–56]. The salient feature is considered as a stimulus that is highlighted. The incongruent condition of MSIT, in which the target response is inconsistent with the target locations, has higher interference and stand out from the congruent one. Accordingly, in MSIT, the activation in right insula may involve in detecting interference and recruiting the interference-resolution.

## Conclusion

In summary, our findings extend the results of prior MSIT studies, confirming that the dACC and prefrontal cortex are the main brain areas activated by MSIT performance. Our meta-analysis confirms cogently, for the first time, two robust activation clusters encompassing the dACC, MPFC, SMA, right IFG, right PUT, and right insula during MSIT performance. Compared to the congruent condition in the MSIT, the incongruent condition is characterized by more conflict and a greater need for cognitive control. On the basis of the functions of the aforementioned brain regions, we postulate that the right insula may send saliently relevant (high interference) signals to the dACC to be used to induce conflict monitoring, and to the SMA, right IFG, and PUT to be used for movement planning and inhibitory control, enabling goal-related flexible, adaptive behavior to be established. Hence, our findings indicate that a cingulate-frontal-striatum network and the right insula may serve as a critical brain circuit in interference resolution.

## Abbreviations

MSIT: multi-source interference task
fMRI: functional magnetic resonance imaging
dACC: dorsal anterior cingulate cortex
CFP: cingulate–frontal–parietal
ES-SDM: effect-size signed differential mapping
MPFC: medial prefrontal cortex
SMA: supplementary motor area
IFG: inferior frontal gyrus
PUT: putamen
preCG: precentral gyrus
ADHD: attention deficit hyperactivity disorder
OCD: obsessive–compulsive disorder
PTSD: posttraumatic stress disorder
